# PDMS-Parylene Hybrid, Flexible Microfluidics for Real-Time Modulation of 3D Helical Inertial Microfluidics

**DOI:** 10.3390/mi9060255

**Published:** 2018-05-23

**Authors:** Bum-Joon Jung, Jihye Kim, Jeong-ah Kim, Hansol Jang, Sumin Seo, Wonhee Lee

**Affiliations:** 1 Graduate School of Nanoscience and Technology, Korea Advanced Institute of Science and Technology (KAIST), Daejeon 34141, Korea; bumjoonjung@kaist.ac.kr (B.J.); xcv4482@kaist.ac.kr (J.K.); jeongah_nano@kaist.ac.kr (J.K.); lookgks@kaist.ac.kr (H.J.); ssm3525@kaist.ac.kr (S.S.); 2 Department of Physics, Korea Advanced Institute of Science and Technology (KAIST), Daejeon 34141, Korea

**Keywords:** inertial microfluidics, cell separation, flexible microfluidics, 3D microchannel

## Abstract

Inertial microfluidics has drawn much attention for its applications for circulating tumor cell separations from blood. The fluid flows and the inertial particle focusing in inertial microfluidic systems are highly dependent on the channel geometry and structure. Flexible microfluidic systems can have adjustable 3D channel geometries by curving planar 2D channels into 3D structures, which will enable tunable inertial separation. We present a poly(dimethylsiloxane) (PDMS)-parylene hybrid thin-film microfluidic system that can provide high flexibility for 3D channel shaping while maintaining the channel cross-sectional shape. The PDMS-parylene hybrid microfluidic channels were fabricated by a molding and bonding technique using initiated chemical vapor deposition (iCVD) bonding. We constructed 3D helical inertial microfluidic channels by coiling a straight 2D channel and studied the inertial focusing while varying radius of curvature and Reynolds number. This thin film structure allows for high channel curvature and high Dean numbers which leads to faster inertial particle focusing and shorter channel lengths than 2D spiral channels. Most importantly, the focusing positions of particles and cells in the microchannel can be tuned in real time by simply modulating the channel curvature. The simple mechanical modulation of these 3D structure microfluidic systems is expected to provide unique advantages of convenient tuning of cell separation thresholds with a single device.

## 1. Introduction

Inertial microfluidics has attracted considerable attention due to its capability of high-throughput microparticle manipulation using simple device structures [1,2,3,4]. The capability to control the position of cells and particles inside of microchannels led to diverse biomedical applications including cell separation and single cell analysis [5,6,7,8,9,10,11,12]. Furthermore, inertial microfluidics allows for extremely high throughput cell separation (>10^6^ cells/min, >1 mL/min), which led to highly efficient circulating tumor cell (CTC) separations and plasma extraction. Among the various inertial microfluidic systems, spiral channels have been recognized by their superior performances owing to the size-dependent Dean drag force which leads to larger separation distances compared to straight channels [4,13,14,15]. However, the understanding of the inertial focusing in spiral channels is still incomplete, and there is no simple guideline for achieving optimal device design and operating conditions for diverse separation targets.

The focusing positions of particles within spiral channels do not follow a simple formula because the relative strength of the inertial lift forces and Dean drag force is a complex function of many parameters including the position in the channel cross-section, flow velocity, channel geometry (curvature, size, and aspect ratio), particle size and particle deformability. In addition, the focusing positions change gradually along the channel because the radius of curvature is not constant with a spiral channel geometry. Due to the difficulty in the exact prediction of the inertial focusing within spiral channels, optimization of the separation efficiency requires empirical determination of the optimal device design and operating conditions for each application. Considering the number of parameters to be tested, it is impractical to find the best device design by varying all possible parameters. Therefore, it is still questionable if the performances of the spiral channels are fully optimized, even though they give reasonably satisfactory results for a particular sample. Moreover, it is extremely inefficient and difficult to find the optimal device design for the separation of individual patient samples that would have different properties than samples used for device optimization. A flexible microfluidic channel that can take a spiral or helical shape in 3D [16] can significantly reduce the efforts for such design optimization process by tuning the channel curvature after device fabrication instead of fabricating multiple devices with different fixed curvatures. 

Although poly(dimethylsiloxane) (PDMS) microfluidic channels have high flexibility, there are several limitations for PDMS to be used as a platform for tunable inertial microfluidics. The high deformability of PDMS causes significant channel cross-sectional enlargement when the internal pressure is induced by fluid flows [17]. The situation becomes even worse if the PDMS channel is constructed as a thin-film structure, where the channel deformation can be as large as several times the original channel size [18,19]. Because the minimum radius of curvature will be limited by the thickness of the microfluidic channels, PDMS channels that require relatively large thickness have a disadvantage as a flexible microfluidic platform. On the other hand, thin-film parylene microfluidics with the film thickness as thin as a few micrometers can provide high flexibility to construct 3D channel structures with enough rigidity to maintain a cross-sectional shape against internal pressure and bending [16]. Thin-film parylene microfluidic devices were fabricated with a newly developed molding and bonding technique using initiated chemical vapor deposition (iCVD) that provides high bonding strength [20]. Nevertheless, there were difficulties in handling of the device and the fabrication of inlets/outlets due to the extremely small thickness of the parylene film. 

Here, we present a PDMS-parylene hybrid microfluidic system with improved convenience in device handling and fabrication. A highly flexible PDMS-parylene hybrid microfluidic device can form a 3D helical structure by simply coiling around a rod. The channel curvature can be easily adjusted to provide tunable inertial focusing and separation of microparticles. We demonstrate real-time tuning of inertial focusing and separation with modulation of the flexible microfluidic 3D structure.

## 2. Materials and Methods

### 2.1. Device Fabrication 

Using conventional photolithography (MA6, Suss MicroTec, Garching, Germany), channel molds were patterned using negative photoresist (SU-8 3050, Microchem, Westborough, MA, USA). Using the SU-8 mold, PDMS molds were replicated. PDMS was spin-coated (Spin Coater ACE-200, Dong Ah Trade Corp., Seoul, Korea) over the SU-8 mold for 60 s at 200 rpm. All PDMS were prepared by mixing the base and cross-linker at a ratio of 10:1. PDMS channels were then cured in a 65 °C oven for >2 h. PDMS to glass and PDMS to PDMS bonding were done by plasma bonding using a plasma cleaner (PDC-002, Harrick Plasma, Ithaca, NY, USA) at high power for 2 min. Parylene C (parylene from here on) was deposited using parylene coater (SCS Labcoater 2, Specialty coating system). Use of ~30 g of Parylene C dimer (DIMER DPX C, Specialty coating system) led to deposition of ~15 μm thick film. To release parylene layer from PDMS mold, the surface of the PDMS mold was treated with oxygen plasma using deep reactive-ion-etch (RIE) process (O_2_, 2.5 cm^3^(STP)/min, 60 W, 60 s) before the deposition of parylene. Such oxygen plasma treatment prevents fusion of parylene with PDMS and allows for the easy detachment of parylene from PDMS.

### 2.2. iCVD

For the bonding of the parylene microfluidic channels, ~200 nm thick layers of epoxy containing polymer, poly(glycidyl methacrylate) (PGMA) were deposited on the oxygen plasma treated parylene layers (O_2_, 2.5 cm^3^(STP)/min, 60 W, 60 s) by iCVD method using glycidyl methacrylate (GMA, 97%, Aldrich, London, UK) monomer and *tert*-butyl peroxide (TBPO, 98%, Aldrich) initiator. The flow rate of GMA and TBPO were set to 4 and 1 cm^3^(STP)/min ratio, respectively. Filament temperature was set to 120 °C and pressure inside the iCVD chamber was to 140 mTorr. Substrates with oxygen plasma treated parylene layer were placed on a cold stage where stage temperature was kept at 30 °C. After PGMA layer was formed, only one of the substrates was reacted with ethylenediamine (EDA). The substrate and EDA were put in a glass Petri dish for 5 min on a 75 °C hot plate. Vaporized EDA reacts with the epoxy group on PGMA surface. Two substrates were then aligned under custom-built aligner and compressed together to form chemical bonding. The substrates were bonded under pressure using a custom-built compressor at an elevated temperature of 85 °C for 8 h. A vacuum oven (350 °C Vacuum Drying Oven, Samheung, Seoul, Korea) was used to remove air bubbles that may be trapped between the substrates and remove oxygen to avoid oxidation damage to parylene.

### 2.3. Materials and Experimental Setup for Inertial Microfluidics

We used various size polystyrene microparticles to observe inertial focusing in flexible microfluidic systems and particle separation demonstration. Green fluorescent particles of size 4.8, 9.9 (Thermo fluoro-max, excitation 468 nm and emission 508 nm), and 15.45 μm (Bangs Laboratories, Inc., Fishers, IN, USA, excitation 480 nm and emission 520 nm), and red fluorescent particles of size 26 μm (Thermo fluoro-max, excitation 542 nm and emission 612 nm) were used. The densities of suspension all microparticles are 1.05 g/cm^3^. NaCl was added to match the densities of the liquid and the particles. In addition, to prevent from particle aggregation 1% of non-ionic surfactant (Tween 20, Sigma-Aldrich, St. Louis, MI, USA) was added to the particle suspension. The particle concentration was in the range of 0.05−0.25 *w*/*v* %. The particle suspension was flowed using a syringe pump (PHD ULTRA CP, Harvard Apparatus, Holliston, MA, USA) with controlling volumetric flow rates. Dynamics of particle migration was observed using an inverted microscope (Eclipse Ti-U, Nikon, Tokyo, Japan) equipped with a digital high-speed camera (Phantom v7.3, Phantom, Wayne, NJ, USA), and a fluorescent illuminator (Intenslight C-HGFIE, Nikon, Melville, NY, USA). Images are analyzed with ImageJ (National Institute of Health, Bethesda, MD, USA).

## 3. Result and Discussion

### 3.1. Parylene-PDMS Flexible Microfluidic System

**Figure 1:**
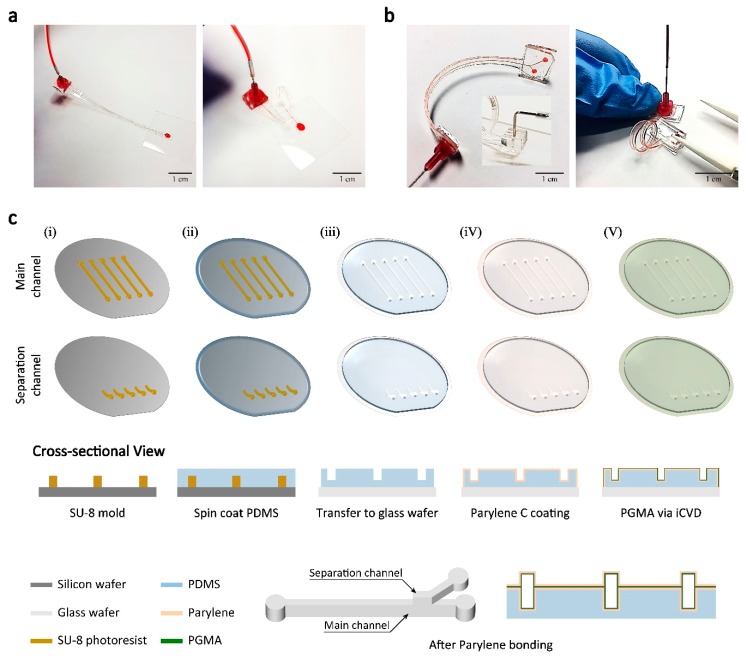
Flexible, thin-film channels based on parylene microfluidics (**a**) Parylene channel built with two 15-µm-thick parylene layers; (**b**) Poly(dimethylsiloxane) (PDMS)-parylene hybrid channel. One of the parylene layers is combined with supportive PDMS layer. Inset: inlet constructed with a piece of PDMS block bonded to supportive PDMS layer; (**c**) Schematics of device fabrication (for PDMS-parylene hybrid channel). The parylene layer is fused with the PDMS layer for the main channel while it is detached from the separation channel mold after initiated chemical vapor deposition (iCVD) bonding.

We developed a fabrication process for a flexible, thin-film microfluidic system using PDMS and parylene (Figure 1). Figure 1a shows a thin-film parylene channel and Figure 1a shows a parylene channel with additional PDMS layer on one side (PDMS-parylene hybrid channel). A PDMS layer with a thickness similar to the channel height can be added to the parylene microfluidic channel for easier handling of the device and fabrication of inlets/outlets connections. These parylene based microfluidic devices can be built with parylene layers that have a few micrometers to tens of micrometer thickness and they can be rolled or curved into various channel geometries. Unlike PDMS devices, they can maintain the channel cross-sectional shape under fairly high pressure owing to the relatively large Young’s modulus of parylene [16]. The channel inlets/outlets can be built by bonding syringe tip with glue [16] or plasma-bond PDMS block onto the PDMS side of the PDMS-parylene hybrid device. 

Figure 1c shows the fabrication process of the flexible microfluidic systems. First, an SU-8 master mold for the main channel and the separation channel were fabricated (i). To create PDMS replica molds, PDMS was spin-coated on the SU-8 molds (ii). Then the PDMS layer was transferred to a transparent glass wafer (iii). The glass wafer was used to allow alignment of the main channel and separation channel. The PDMS layer was permanently plasma-bonded when it was used as a mold for the parylene channel. When fabricating PDMS-parylene hybrid channel, in order to leave PDMS layer on one side of the channel, an additional thin PDMS layer was spin-coated on a glass wafer before PDMS mold transfer. The PDMS replica from the SU-8 mold was plasma bonded to the thin PDMS layer instead of the glass wafer so that the bonded PDMS layers can be detached with a parylene layer from the glass wafer. Then, ~15 μm thickness parylene layers were deposited on the substrates with PDMS layers (iv). Normally, parylene deposited on PDMS cannot be detached because parylene monomer can penetrate into PDMS body and the polymerized parylene fuses with PDMS [21]. To detach the parylene layer from the PDMS mold, the surface of the PDMS was treated with oxygen plasma before parylene deposition process. Although the exact mechanism is unknown, the change in the PDMS surface energy seems to prevent the parylene monomer penetration and limit the polymerization to occur only on the surface. For fully parylene channels, both PDMS molds would be treated with oxygen plasma. Only the PDMS mold for separation channel was treated with oxygen plasma to create PDMS-parylene hybrid microchannel. To form a microfluidic channel, two parylene layers on the PDMS molds were bonded with PGMA nano adhesive layer (~200 nm) deposited by iCVD technique (v) [20]. After PGMA layers were deposited, one side of a substrate was reacted with vaporized EDA to form amine groups. The substrates were then aligned using custom-built aligner and bonded together. Two substrates can be bonded with strong chemical bonding between epoxy and amine groups, which results in high bonding strength (~25 bar) [20]. After the bonding process, an inlet and outlets were constructed using syringe tips, pieces of PDMS, and diced slide glasses. The inlet and outlets were punched through the thin-film channels. Then, the diced slide glass was bonded with O_2_ plasma on the PDMS side of the channel to cover up the inlet and outlet holes and the syringe tip is assembled on the opposite side with epoxy glue. Channel inlet and outlets can also be constructed with the piece of PDMS block instead of syringe tip (see inset Figure 1b). First, the diced slide glass was glued on the parylene side. Then the inlet and outlets were punched from the PDMS side of the channel. Finally, small pieces of PDMS blocks with inlet and outlet holes were plasma bonded to the supportive PDMS layer of the PDMS-parylene hybrid channel. The PDMS inlet allows easy assembly with tubing, while the syringe tip inlet provides tighter tubing connections. 

### 3.2. Operation of the 3D Helical Inertial Microfluidic System

**Figure 2:**
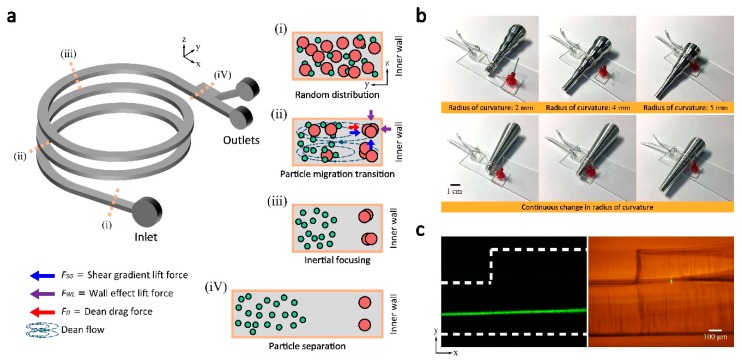
Flexible 3D helical inertial microfluidics operations. (**a**) Schematic illustration of inertial focusing in a helical microchannel. Stable focusing positions are formed near the inner wall corners by the balance of inertial lift forces and Dean drag force. Small particles do not focus and circulate within the core of Dean vortices; (**b**) Control of radius of curvature, *R*. PDMS-parylene hybrid channels are coiled on aluminum rods, with stages of distinct diameters and continuously changing diameter. (**c**) Side view observation of the channel at the junction of the main channel and separation channel in fluorescent and bright field images.

Figure 2a represents the schematic image of the helically coiled Parylene channel as well as the illustration of particle migration mechanism inside the microfluidic channel. We defined the coordinate system matching the channel cross-section following the convention of other research works for spiral inertial microfluidics: *x*-direction aligned with the main flow directions, *y*-direction in the width direction and *z*-direction in the height direction. Note the channel height direction and width direction is switched before and after the flexible channel coiling. In a curved channel, as shown in Figure 2a, the secondary flows, namely, Dean flows are induced by the uneven fluid inertia due to the difference in fluid velocity between the center and wall of the channel [1,2,22,23]. The magnitude of the Dean flow is known to be characterized by the dimensionless Dean number (*De*)

*De* = (*ρ_f_UH*/*μ_f_*)(*H*/2*R*)^1/2^*= Re*(*H*/2*R*)^1/2^ (1)

and Dean drag force (*F_D_*) [24,25] is

*F_D_* = 5.4 × 10^−4^π*μ_f_De*^1.63^*a_p_* (2)

where *ρ_f_* is the fluid density, *U* is the average fluid velocity, *H* is the hydraulic diameter, *μ_f_* is the fluid viscosity, *R* is the radius of channel curvature, and *a_p_* is the diameter of the particle. The Dean drag force in addition to inertial lift forces (shear gradient lift force and wall effect lift force) is known to enhance efficiency and throughput for size-based microparticle separations in spiral channels. For relatively larger particles, focusing positions form near the inner wall where wall effect lift force equilibrates opposing shear gradient lift force and Dean drag force. While larger particles have stable focusing positions, smaller particles may be entirely dominated by Dean drag force and eventually get trapped in the core of Dean vortices [4]. Since the magnitude of *F_D_* is a function of the channel curvature, the ability to control the radius of curvature suggest an important route to manipulate and cells and particles in spiral inertial microfluidics. 

To easily adjust the radius of curvature of the 3D microfluidic channels, the flexible, thin-film microfluidic devices are coiled around rods with varying diameter. Figure 2b shows the two types of rods built with Al: a rod with multiple stages with different diameter and a rod with a continuously changing diameter (Figure 2b). By sliding flexible PDMS-parylene hybrid channel along the Al rod, the channel curvature can be easily adjusted, by which allows convenient tuning of the Dean flow as well as the focusing position of the particles without switching devices. As shown in Figure 2b, the Al rod with multiple stages has six different diameters; 2, 3, 4, 5, 6, and 7 mm. For a demonstration of the real-time tuning of the focusing position, the rod with continuously changing diameter was used. 

Figure 2c show the separation channel connection to the main channel. Unlike 2D spiral channels, side-view observation at the separation channel is necessary for the 3D coiled helical channel. Bright-field and fluorescent microscopy pictures in the side-view show clear images of the device and particle trajectory at the junction of the main channel and separation channel.

### 3.3. Size-Dependent Particle Focusing with Varying Radius of Curvature

**Figure 3:**
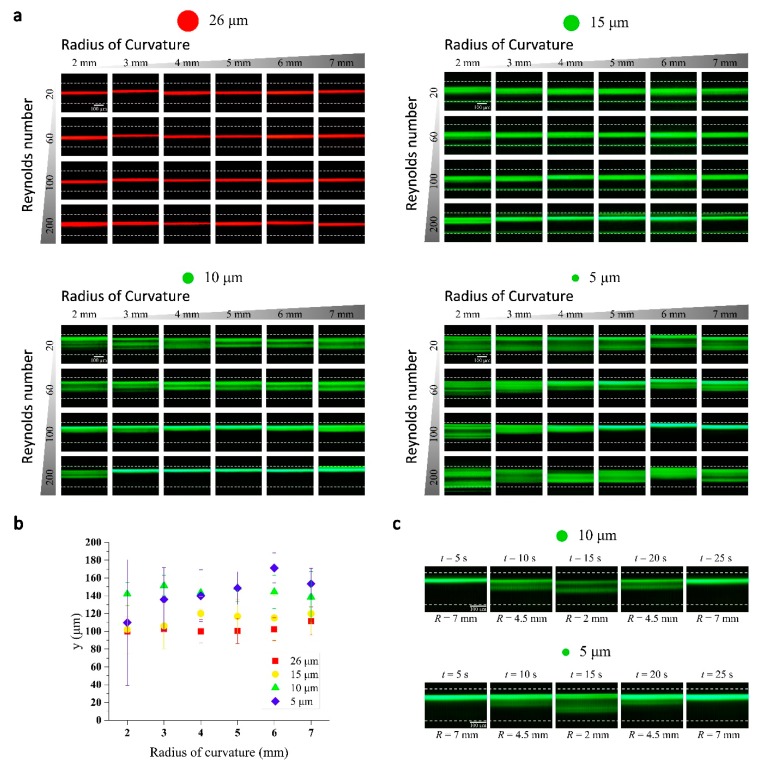
Inertial focusing in the 3D helical channel and real-time tuning. (**a**) Inertial focusing of particles with 4 different sizes (*a_p_* = 5, 10, 15, and 26 µm). The fluorescent images of particle streaks collected with a varying radius of curvature and flow rate (*Re*); (**b**) full width at half maximum (FWHM) of the fluorescent streak images vs. radius of curvature is plotted for *Re* = 100; (**c**) Snapshots of the real-time modulation of inertial focusing in a flexible PDMS-parylene hybrid channel. (Video S1 and S2).

As shown in Figure 3a, inertial focusing of microparticles in the 3D helical channel was tested with four different sized particles (5, 10, 15, and 26 µm in diameter). The flexible microfluidic channel had a dimension of 100 μm × 200 μm × 60 mm (width × height × length). The cross-sectional dimensions are small compared to typical spiral inertial microfluidic channels. It was chosen to show the small radius of curvature of the flexible 3D channel can induce strong enough Dean flows even with the small cross-section. The measurement was performed with six different radius of curvatures (2, 3, 4, 5, 6, and 7 mm) and four different combinations of flow rates (*Re* = 20, 60, 100 and 200). The inertial focusing of the particles in the flexible 3D channel shows both similar and different results that have been observed with 2D spiral channels. For relatively larger 26 and 15 µm particle streaks appear narrow in a wide range of *Re* and *R* where 10 and 5 µm particles appear as rather wide bands. The 26 and 15 µm particles’ equilibrium positions were mostly located near the center of the channel (in *y*-direction) instead of the position close to the inner wall which normally observed in 2D spiral channels. We believe such focusing near the channel center is due to relatively small channel dimensions. In a spiral channel with a small cross section, inertial lift forces can completely dominate over Dean drag force and the focusing near the center of long channel faces is expected, similar to the case of low aspect ratio straight channels. The relative strength of the Dean drag compared to inertial lift force increases in a channel with larger cross-sectional dimensions, and the focusing positions for similar conditions (*Re*, *R* and *a_p_*) were observed shifting towards the inner wall (Figure S1), which more closely resembles the focusing in 2D spiral channels. In general, Dean flow effects seem weak at low *Re* and, with the increase of *Re*, the focusing position shifted towards the inner wall showing the Dean drag become strong enough to change the focusing positions. With the decrease of channel curvature, the Dean number increases and the shift of focusing towards the inner wall is expected to be observed. However, the general results (especially, for *a_p_* = 15 µm, *Re* = 200) show that the focusing position shift with Dean number change is opposite to what is predicted. Currently, we do not understand the mechanism of this unexpected results. 

Inertial lift forces are strong function of particle size and it is expected that focusing to a narrow stream becomes difficult with the smaller 10 and 5 µm particles. On the other hand, the Dean drag effects become more evident with smaller size particles, therefore the focusing positions shift towards the inner wall and the peaks broaden. Interestingly, two or more fluorescence streak lines, instead of broad bands, were found in some cases. Our helical channel have ~3 mm displacement between each turn. It has been reported that the channel translation in helical channel causes a break of symmetry in the two symmetrical Dean flow vortices [26]. We believe such changes cause asymmetric force balances in the different Dean vortices, which leads to two different focusing position in *y*-direction for different *z* positions. As discussed previously, the inertial focusing in spiral channels is a complex problem involving multiple parameters. One to one comparison of the current results with previous research results will be difficult. Moreover, the helical channel adds another complexity with asymmetric Dean flows. However, it is clear that the flexible microfluidic system provides an easy method to investigate the inertial focusing results. In addition, the unexpected results arising from helical channel will be an interesting system to investigate further. Figure 3b summarizes the representative focusing trend at *Re* = 100; the center and the full width at half maximum (FWHM) of the fluorescent streaks are shown with varying *R*.

We have demonstrated the real-time tuning of inertial focusing by modulation of 3D channel curvature. The radius of curvature of the 3D helical channel can be adjusted using the rod shown in Figure 2b, while fluid flow is set to a constant value (*Re* = 100). The change in channel curvature leads to changes in the magnitude of the Dean flow that affect the inertial focusing. The fluorescent intensity profiles look similar to the result shown in Figure 3a. There exists small discrepancy, which is due to the 3D channel curvature is not strictly constant with the coiling rod that has continuously varying diameter. Nevertheless, this simple demonstration clearly shows that the inertial focusing can be significantly changed with a mechanical modulation of 3D channel geometry.

### 3.4. Tuning of Inertial Separation 

**Figure 4:**
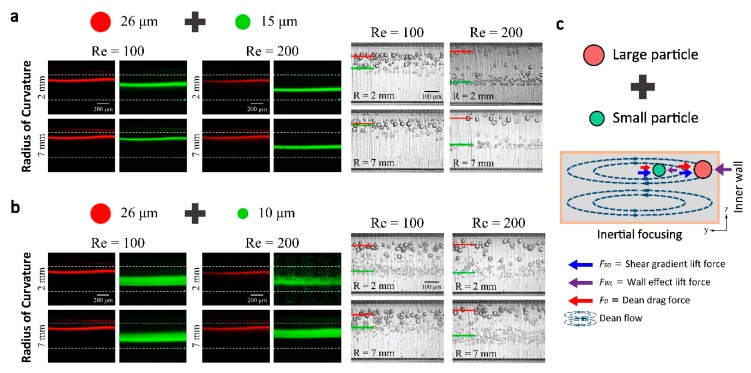
Demonstration of particle separation. The mixed particle suspension flowed and particle distributions were observed near the outlet for particle mixture of (**a**) 26 and 15 μm and (**b**) 26 and 10 μm. Fluorescent streak images on the left and the stacked high-speed images (200 images) on the right. The inertial focusing and the separation distance can be controlled with the flow rate and the radius of curvature; (**c**) Separation principle based on force balance between inertial lift forces and Dean drag force.

In general, microfluidic particle separation techniques can be classified as a passive and active technique [27] (pp. 1–14). The inertial focusing as passive separation technique has many advantages. The capability to modulate the radius of curvature with the flexible 3D microfluidics platform adds advantages of active tuning of separation efficiency and separation size threshold. We have demonstrated the feasibility of the tuning of particle separation efficiency by adjusting 3D coiling radius of a single device rather than using multiple devices with a different design. Two different combinations of particle mixtures (26 and 15 µm particles and 26 and 10 µm particles) were separated in different flow rate and radius of curvature conditions. The separations results were achieved with 400 µm × 100 μm (height × width) channel because it is known, for spiral channels, such low aspect-ratio rectangular channels have an advantage of the larger effect of Dean drag force and maximized separation distance between different sized particles. The inertial focusing of individual particles within the 400 µm × 100 μm channel can be found in the supplementary information (Figure S1). By adjusting the radius of curvature from 2 mm to 7 mm, it is shown in Figure 4 that the separation distance between two different size particles has increased. Most dramatic change of a focusing position appeared to be at a flow rate at *Re* = 100. On the other hand, at a flow rate of *Re* = 200, the gap distance between two different sized particles was maintained, but there was no significant difference when the radius of curvature was changed. Figure 3c, shows a basic principle of size-dependent particle separations in spiral inertial microfluidic channels. Although focusing position changes with particle size change would not as simple as the illustration, the major source of the different force balance is Dean drag force difference that is pushing the particles towards the inner wall near the top and bottom channel wall. Larger the particle becomes the larger Dean drag, which can be balanced by larger wall effect lift force near the inner wall. 

### 3.5. 3D Parallelization of Spiral Inertial microfluidic Channel

**Figure 5:**
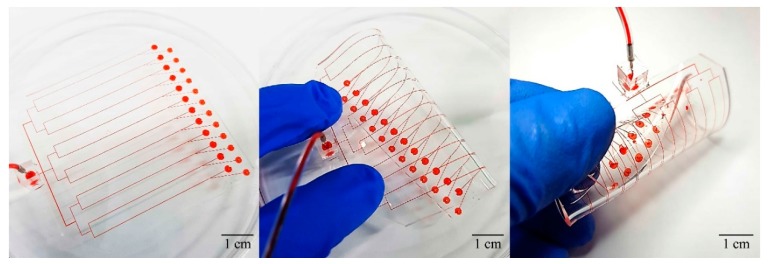
Parallelization of the 3D spiral channel can be achieved easily by rolling the 2D parallel channels.

Flexible microfluidic systems have the additional benefit of easy parallelization of 3D channels compared to other fabrication methods such as 3D printing [6] and selective etching after femtosecond laser irradiation [28]. Many microfluidic devices can be easily designed to have parallel channels to provide increased throughput. However, it is difficult to parallelize spiral inertial microfluidic devices because the spiral channel shape makes it difficult to connect the inlets/outlets of multiple channels. An alternative method would be stacking the channels in the height direction but it requires complicated tubing connection between the stacks, which is highly impractical. As shown in Figure 5, flexible microfluidics allows simple, straightforward parallelization of spiral channels; a row of spiral channels connected to a single inlet can be constructed from 2D parallel channels. The parallelized device has 12 identical channels in the current design. It may be designed to have different channel dimensions for different separation threshold or efficiency. This novel method of 3D parallelization would inspire new approaches for compact 3D microfluidic systems for large-scale integration.

## 4. Conclusions

Inertial focusing is highly dependent on the channel geometry and structure, therefore, by modulating 3D channel structure, flexible microfluidic channels can provide active tuning capability to inertial particle separation that is passive separation technique in nature. In this work, we presented a flexible, thin-film PDMS-parylene microfluidic systems. The fabrication of parylene channel is based on the previously developed bonding and molding technique based on iCVD bonding. PDMS-parylene hybrid channel fabrication is enabled by the control of adhesion between the parylene layers and the PDMS layers. The PDMS-parylene hybrid channel allows easy handling and fabrication of the inlets/outlets. The thin-film PDMS-parylene channels allow real-time modulation of inertial focusing with adjustable 3D structure by providing high flexibility while maintaining uniform cross-sectional shape under high internal pressures. The 3D helical channel has a constant radius curvature throughout the channel and the high flexibility and the small channel thickness allow inertial particle focusing in shorter channels lengths than 2D spiral channels due to high Dean numbers. Most importantly, tuning of the inertial focusing can be done simply by modulating the 3D channel curvature. The capability of simple mechanical modulation of 3D channel structure can reduce the efforts for optimization of cell separation efficiency by eliminating the needs for the redesign of the channel, which will be highly beneficial for liquid biopsy applications such as CTC separations. We observed the inertial focusing in the 3D helical channel while varying the radius of curvature and flow rate using a single device. We also demonstrate the real-time tuning of the inertial focusing and feasibility of microparticle separation in a 3D helical channel. In addition, the flexible microfluidic system can provide a simple method of parallelization of the spiral channels in 3D. We anticipate the flexible, thin-film microfluidic system will lead to diverse methods for constructing unconventional 3D structures that may allow novel functionality with real-time tunability.

## Supplementary Materials

The following are available online at http://www.mdpi.com/2072-666X/9/6/255/s1, Video S1: Real-time tuning of inertial focusing (5 micron), Video S2: Real-time tuning of inertial focusing (10 micron).
