# Supplementary figures and images for: PDMS-Parylene Hybrid, Flexible Microfluidics for Real-Time Modulation of 3D Helical Inertial Microfluidics
Source: Micromachines (Basel). 2018 May 23;9(6):255. doi: 10.3390/mi9060255 (PMC6187561; doi:10.3390/mi9060255)

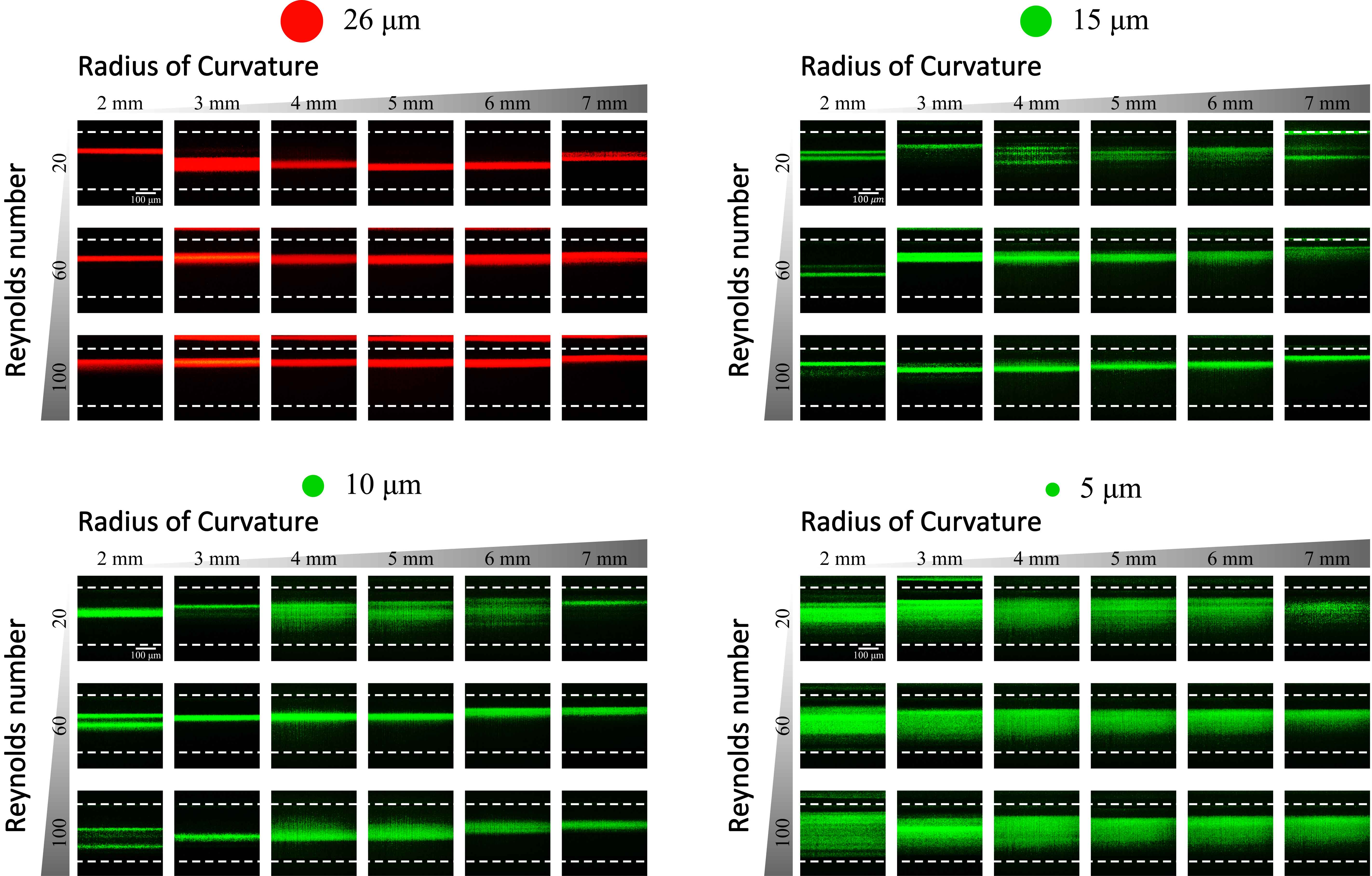

Supplement: Supplementary file 1 [file micromachines-09-00255-s001.zip › supplementary/Figure S1.jpg]
